# Investigating cortical activity during cybersickness by fNIRS

**DOI:** 10.1038/s41598-024-58715-2

**Published:** 2024-04-06

**Authors:** Sang Seok Yeo, Seo Yoon Park, Seong Ho Yun

**Affiliations:** 1 Department of Physical Therapy, College of Health and Welfare Sciences, Dankook University, Cheonan, Republic of Korea; 2 Department of Physical Therapy, College of Health and Welfare, Woosuk University, Wanju, Republic of Korea; 3 Department of Public Health Sciences, Graduate School, Dankook University, Cheonan-si, Republic of Korea

**Keywords:** Cybersickness, Functional near infrared spectroscopy, Virtual reality, Angular gyrus, Cortex, Sensory processing, Visual system, Biomarkers

## Abstract

This study investigated brain responses during cybersickness in healthy adults using functional near-infrared spectroscopy (fNIRS). Thirty participants wore a head-mounted display and observed a virtual roller coaster scene that induced cybersickness. Cortical activation during the virtual roller coaster task was measured using fNIRS. Cybersickness symptoms were evaluated using a Simulator Sickness Questionnaire (SSQ) administered after the virtual rollercoaster. Pearson correlations were performed for cybersickness symptoms and the beta coefficients of hemodynamic responses. The group analysis of oxyhemoglobin (HbO) and total hemoglobin (HbT) levels revealed deactivation in the bilateral angular gyrus during cybersickness. In the Pearson correlation analyses, the HbO and HbT beta coefficients in the bilateral angular gyrus had a significant positive correlation with the total SSQ and disorientation. These results indicated that the angular gyrus was associated with cybersickness. These findings suggest that the hemodynamic response in the angular gyrus could be a biomarker for evaluating cybersickness symptoms.

## Introduction

Motion sickness is a condition in which symptoms such as nausea, vomiting, and dizziness appear singly or in combination that occur in situations where acceleration and deceleration are repeated^1,2^. The human nervous system maintains balance and perceives body movements based on vestibular, visual, and somatosensory information^3,4^. For instance, if vision is fixed on one place when speed changes while moving in a boat or car, the brain receives conflicting vestibular and visual information, which can cause motion sickness^2,5^. The degree of motion sickness varies from person to person and is determined by family history, nervous system diseases, sensitivity to movement, and visual stimulation^5^.

Cybersickness symptoms include nausea, dizziness, headache, and paresthesia that occur when using immersive virtual reality, such as head-mounted displays, which are symptoms similar to those of general motion sickness^6,7^. Cybersickness is also caused by a discrepancy between visual information in the virtual reality and vestibular and somatosensory information, various changes in visual information, and relatively little physical movement^7^. The sensory conflict theory explains that motion sickness and spatial disorientation can occur because of conflicts between sensory inputs related to spatial orientation and the perception of movement^8,9^. When there is a discrepancy or conflict between sensory inputs, the human brain recognizes it as a threat or an abnormal situation and induces symptoms such as dizziness and nausea as a protective response^8,9^. These problems can be a challenge in the development of virtual reality technology, particularly in the application of virtual reality rehabilitation technology for patients with neurological diseases such as stroke.

Along with the development of virtual reality technology, various methods have been developed to minimize cybersickness through improved hardware, software optimization, and design technologies that more effectively align visual and vestibular signals^10–12^. A recent study reported that cybersickness was reduced by simultaneous auditory stimulation and actual movement under virtual reality conditions^12^. This is considered to be the result of preventing sensory conflicts by providing appropriate auditory and somatosensory stimulation in accordance with the visual stimulation in virtual reality^12^. The functional neuroimaging studies investigated the how the brain regions respond cybersickness in the virtual reality^13–15^. Electroencephalography (EEG) studies reported that cybersickness was associated with increased spectral power in delta, theta, and alpha frequency bands through frequency and time–frequency spectral analysis^13,14^. However, EEG is susceptible to motion artifacts and electrical signal interference when interacting with virtual reality technology^16^. Among functional neuroimaging techniques, the functional near-infrared spectroscopy (fNIRS) has advantage of being less susceptible to motion artifacts and electrical noises and higher spatial resolution than EEG^16^. It is a non-invasive optical method that indirectly detects cortical activity based on hemodynamic response and is considered a promising neuroimaging technique for the virtual reality tasks^17^. Previous fNIRS study reported that individuals who experienced cybersickness symptoms exhibited an increase in the concentration of oxyhemoglobin (HbO) in the parieto-temporal regions^15^. In addition, HbO concentration showed positive correlation with nausea and motion sickness symptoms. However, this study was pilot study based on a small sample size which the results should be interpreted with caution. In addition, they mainly investigated the nausea using 10-point scale among cybersickness symptoms. Given that cybersickness symptoms include nausea, oculomotor discomfort, and disorientation, it is necessary to employ more comprehensive assessment instrument. The subjective sickness questionnaire (SSQ) is the most widely used for measuring the subjective level of cybersickness^18^. It consists of sixteen items associated with cybersickness and employs a straightforward scoring approach to evaluate the severity of discomfort (0: no symptom; 1: mild; 2: moderate; 3: severe)^19^. The SSQ has been demonstrated to be reliable and is currently regard as the gold standard assessment tools for evaluating comprehensive symptoms of cybersickness^20^.

Therefore, the purpose of this study was to investigate the changes in cerebral cortex activation and cybersickness symptoms in virtual reality using the fNIRS and SSQ.

## Methods

### Participants

Thirty healthy adults (17 men and 13 women; mean age: 24.17 ± 3.37 years; dominant hand: right) were recruited for this study. None of the participants had a history of musculoskeletal, neurological, or psychiatric disease. The study was conducted in accordance with the relevant guidelines and regulations of the Declaration of Helsinki. The study protocol was approved by the Institutional Review Board of Dankook University (DKU 2023-01-016-001). All participants were given detailed instructions regarding the experiment, and they provided written informed consent to participate in the study.

### Measurements

#### Functional near infrared spectroscopy (fNIRS)

**Figure 1:**
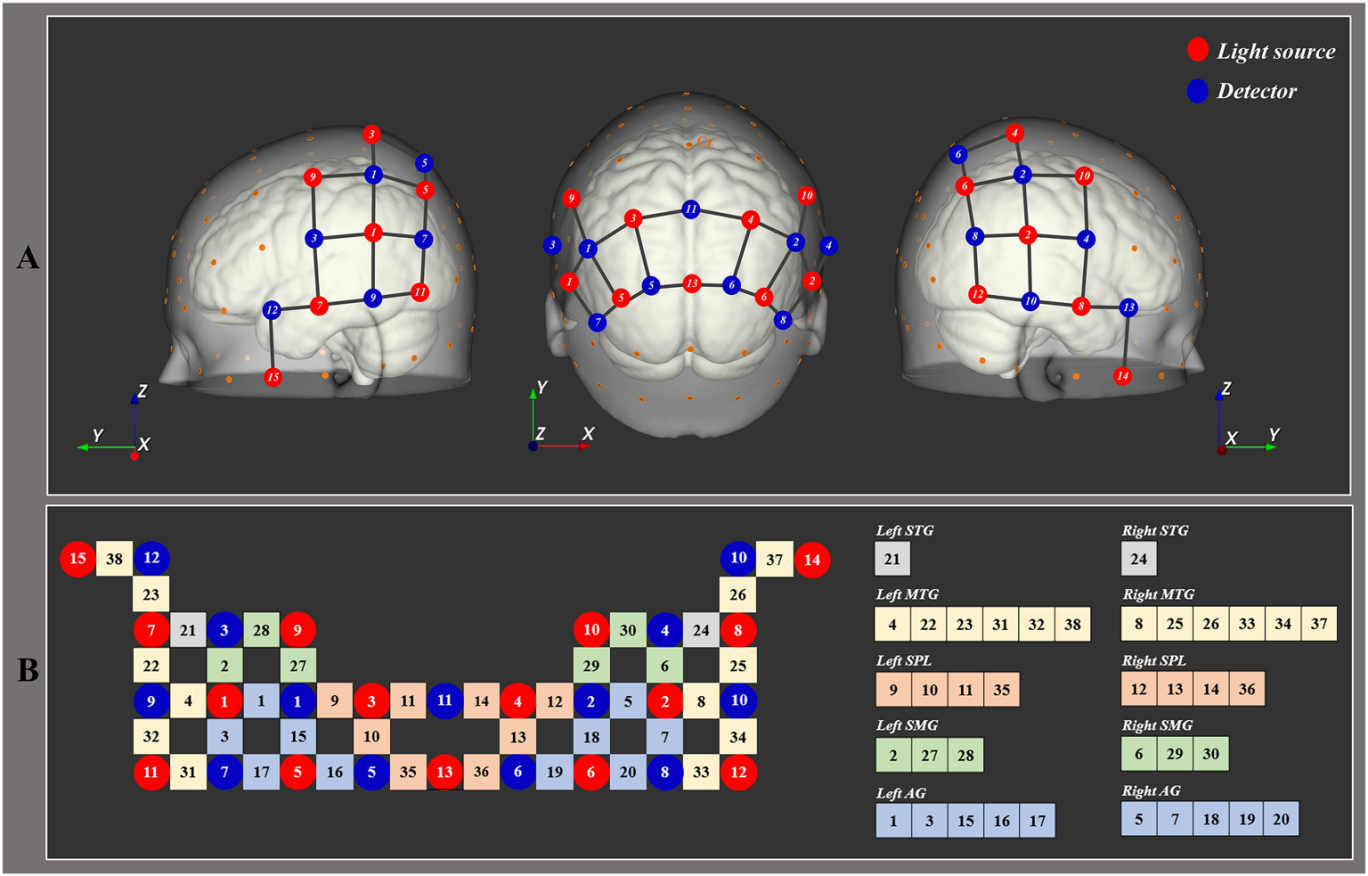
fNIRS optode placement and channel configuration. (**A**) fNIRS optode placement; the fifteen red and thirteen blue circles represent the positions of the light source and detectors, respectively. (**B**) Channel configuration and region of interest (ROI); *STG* superior temporal gyrus, *MTG* middle temporal gyrus, *SPL* superior parietal lobule, *SMG* supramarginal gyrus, *AG* angular gyrus.

fNIRS data were acquired using the continuous-wave Nirsport 2 (Nirx Medical Technologies LLC, Berlin, Germany) with a sampling rate of 12.52 Hz. The optodes were positioned on the cap in accordance with international 10–20 systems using NIRSite software (NIRx Medical Technologies, LLC, Los Angeles, CA, USA) and the fNIRS Optodes’ Location Decider toolbox. We employed 15 light sources and 13 detectors to record the optical light intensity at two wavelengths (760 and 850 nm). The light source and detector arrangements covered a total of 38 channels for data acquisition. Previous studies reported that temporoparietal junction and parieto-insular vestibular cortex play an important role in vestibular processing, proprioception processing, and the multisensory integration associated with cybersickness^21,22^. Given these findings, the regions of interest were the bilateral superior temporal, middle temporal, superior parietal, supramarginal, and angular gyri (Fig. 1).

#### Simulator Sickness Questionnaire (SSQ)

Cybersickness symptoms were measured using a Simulator Sickness Questionnaire (SSQ). The SSQ is a self-report questionnaire designed to evaluate symptoms associated with simulator sickness. It consists of 16 self-reported items related to cybersickness symptoms, such as dizziness, headache, and eye strain, scored using a four-point Likert scale (0 = none, 1 = mild, 2 = moderate, and 3 = severe). The total SSQ score and three subscale scores for nausea, oculomotor distress, and disorientation are calculated according to a specific scoring procedure^23^. The SSQ exhibited high internal consistency with a Cronbach’s alpha of 0.868^24^.

### Procedure

Before the experimental session, the participants were given a 10-min explanation of the experiment and completed an SSQ and a demographic questionnaire to familiarize themselves with the laboratory environment. Each participant was equipped with head-mounted display virtual reality (Oculus Quest 2, Meta, Menlo Park, CA, USA) and fNIRS devices. The experimental session consisted of three-block paradigm. Each block included: a 30-s of rest; a task phase, lasting 120-s; and a recovery phase, lasting 30-s. There is currently no gold standard for the number of blocks to reduce variability of fNIRS signal^25^. Nevertheless, previous studies reported that employing at least three blocks enables the averaging of fNIRS signals and reduces anticipatory contributions^26^. Participants were asked to fixate on a cross in the center of a black screen while rest phase for 30 s. Then, each participant was instructed to observe a virtual roller coaster scene during the 120 s task. The duration of the virtual reality exposure was determined based on the previous studies investigating cybersickness^27,28^. The sound of the virtual roller coaster scene was not provided to measure visually induced cybersickness. In the 30 s recovery phase, participants were instructed to fixate on a cross in the center of a black screen. Participants self-reported the severity of their cybersickness symptoms using an SSQ questionnaire following the experiment session.

### Data analyses

The fNIRS data were analyzed using nirsLAB version 2019.04 (NIRx Medical Technologies LLC, Berlin, Germany). The signal quality of each channel was evaluated using the coefficient of variation (CV = standard deviation/mean), with a level of 15% or less regarded as adequate. The data were preprocessed by removing discontinuities and spike artifacts. Discontinuities were automatically detected and removed (std threshold = 5)^29^. Spike artifacts, which were confirmed by two independent researchers, were replaced with random signals (random numbers that were sampled from a Gaussian distribution, with a standard deviation equal to the average of the 4 s time intervals preceding and following the motion artifacts, and with a mean equal to the data value)^30^. Then, the data were filtered through a bandpass filter (0.001–0.20 Hz) with a 15% roll width to eliminate the effects of heartbeat, respiration, and low-frequency signal drifts for each wavelength^30^. Optical density was converted to oxyhemoglobin (HbO), deoxyhemoglobin (HbR), and total hemoglobin (HbT) concentrations using the modified Beer–Lambert Law^31,32^.

We performed the Statistical Parameter Mapping NIRS-SPM (SPM 8) tool for topographical analysis. A general linear model (GLM) with a canonical hemodynamic response curve (HRF) was used to analyze significant task-related cortical activation separately for HbO, HbR, and HbT for each individual^31^. At the individual level, a SPM-1 analysis was performed to estimate the degree of activation for each channel. In the SPM-1 analysis, a canonical HRF was considered, and pre-whitening was omitted. This was followed by application of Gaussian full width at half maximum 4 model and discrete cosine transform temporal parameter with a high-pass period cutoff of 128 s. Then, GLM were obtained for each individual based on the HbO, HbR, and HbT signals. The design matrix was set up to contrast the rest (0)/task (1)^33^. For the multiple data analysis, a SPM-2 analysis was performed. SPM-1 and SPM-2 t-maps were conducted based on those t-contrasts with p < 0.05. p-values were corrected using the false discovery rate (FDR) to control for false positives in multiple comparisons. In the significant channels, the beta-coefficient of HbO, HbR, and HbT in each channel was extracted from the GLM. The beta-coefficient, representing the amplitudes of the hemodynamic responses, indicates the intensity of cortical activation^34^. To evaluate the relationship between cortical activity and cybersickness symptoms, Pearson correlations with FDR correction between the SSQ score data and beta coefficients of HbO, HbR, and HbT in each channel were performed using the SPSS software (version 21.0; IBM Corp. Armonk, NY, USA).

## Results

### SSQ

The results of the descriptive statistics for the SSQ scores are as follows: total SSQ (73.46 ± 50.56), nausea (54.86 ± 52.86), oculomotor distress (53.06 ± 33.20), and disorientation (95.95 ± 67.70).

### Group analysis of HbO, HbR and HbT values

**Figure 2:**
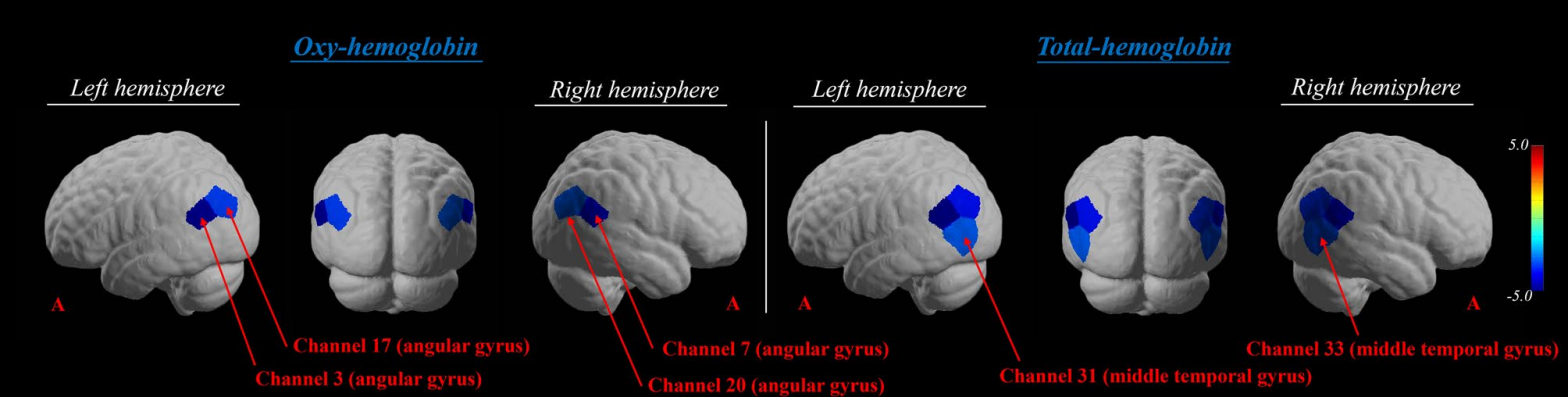
Group-average t-statistic maps of oxyhemoglobin and total hemoglobin values during cybersickness using NIRSLab software (*p*_*corrected*_ < 0.05).

**Figure 3:**
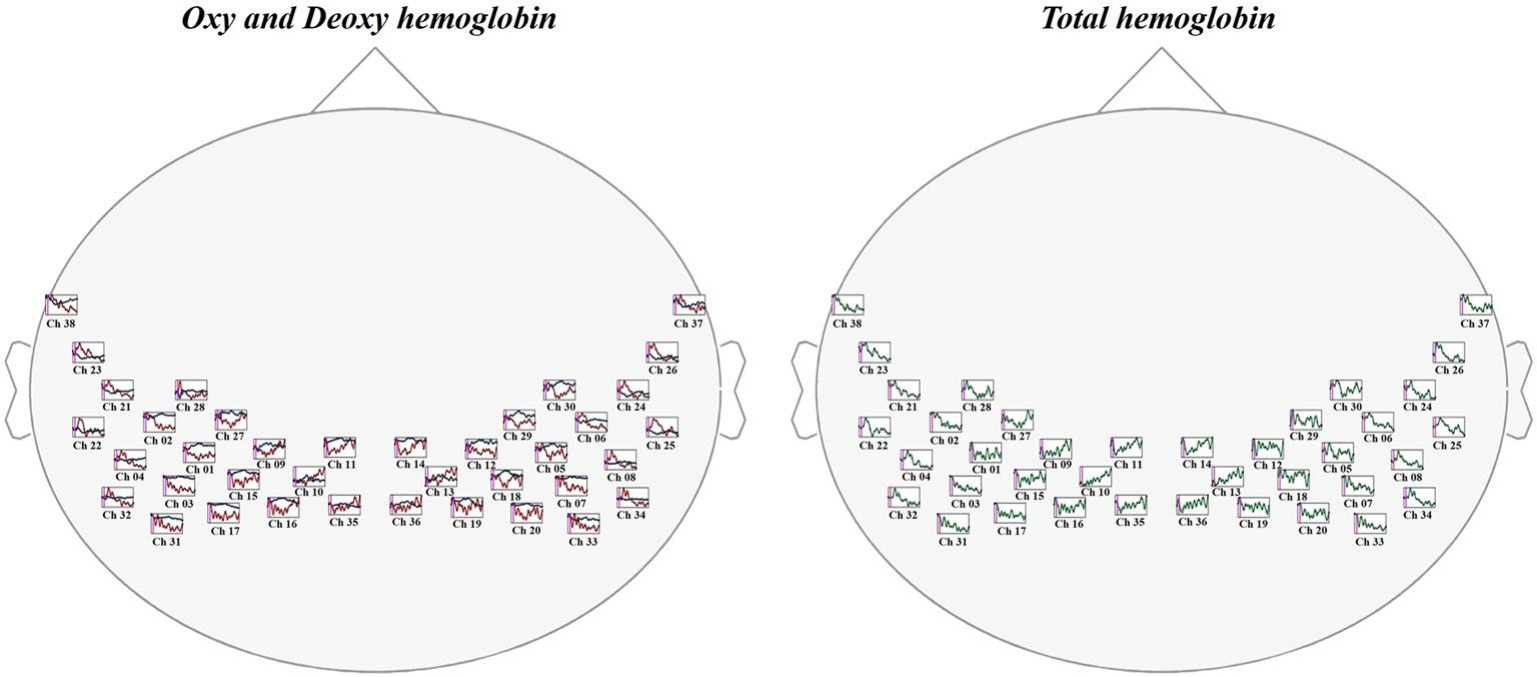
Time course of hemodynamic responses. The red, blue, and green lines represent oxyhemoglobin, deoxyhemoglobin, and total hemoglobin, respectively.

In the group analysis, HbO values showed significant deactivation in the bilateral angular gyrus with respect to resting (*p*_*corrected*_ < 0.05). There was no significant activation or deactivation in the group analysis of HbR values (*p*_*corrected*_ > 0.05). The HbT values revealed significant deactivation in the bilateral angular and middle temporal gyri (*p*_*corrected*_ < 0.05) (Table 1 and Fig. 2). Figure 3 showed the time course of hemodynamic responses of HbO, HbR, and HbT.

**Table 1:** Significant channels for HbO and HbT during cybersickness.

Brain region	Channel	t	*p*_*uncorrected*_	*p*_*corrected*_
HbO				
Lt angular gyrus (BA 39)	3	− 4.73	< 0.001*	0.001*
	17	− 3.15	0.002*	0.023*
Rt angular gyrus (BA 39)	7	− 3.85	< 0.001*	0.006*
	20	− 2.85	0.004*	0.037*
HbT				
Lt angular gyrus (BA 39)	3	− 6.04	< 0.001*	< 0.001*
	17	− 3.71	< 0.001*	0.006*
Lt middle temporal gyrus (BA 21)	31	− 2.85	0.004*	0.025*
Rt angular gyrus (BA 39)	7	− 4.18	< 0.001*	0.002*
	20	− 3.35	< 0.001*	0.010*
Rt middle temporal gyrus (BA 21)	33	− 3.07	< 0.002*	0.017*

*p*_*corrected*_ the p-value was corrected using false discovery rate, *BA* Brodmann area, *HbO* oxyhemoglobin, *HbT* total oxyhemoglobin.

### Relationship between cortical activity and cybersickness symptoms

**Figure 4:**
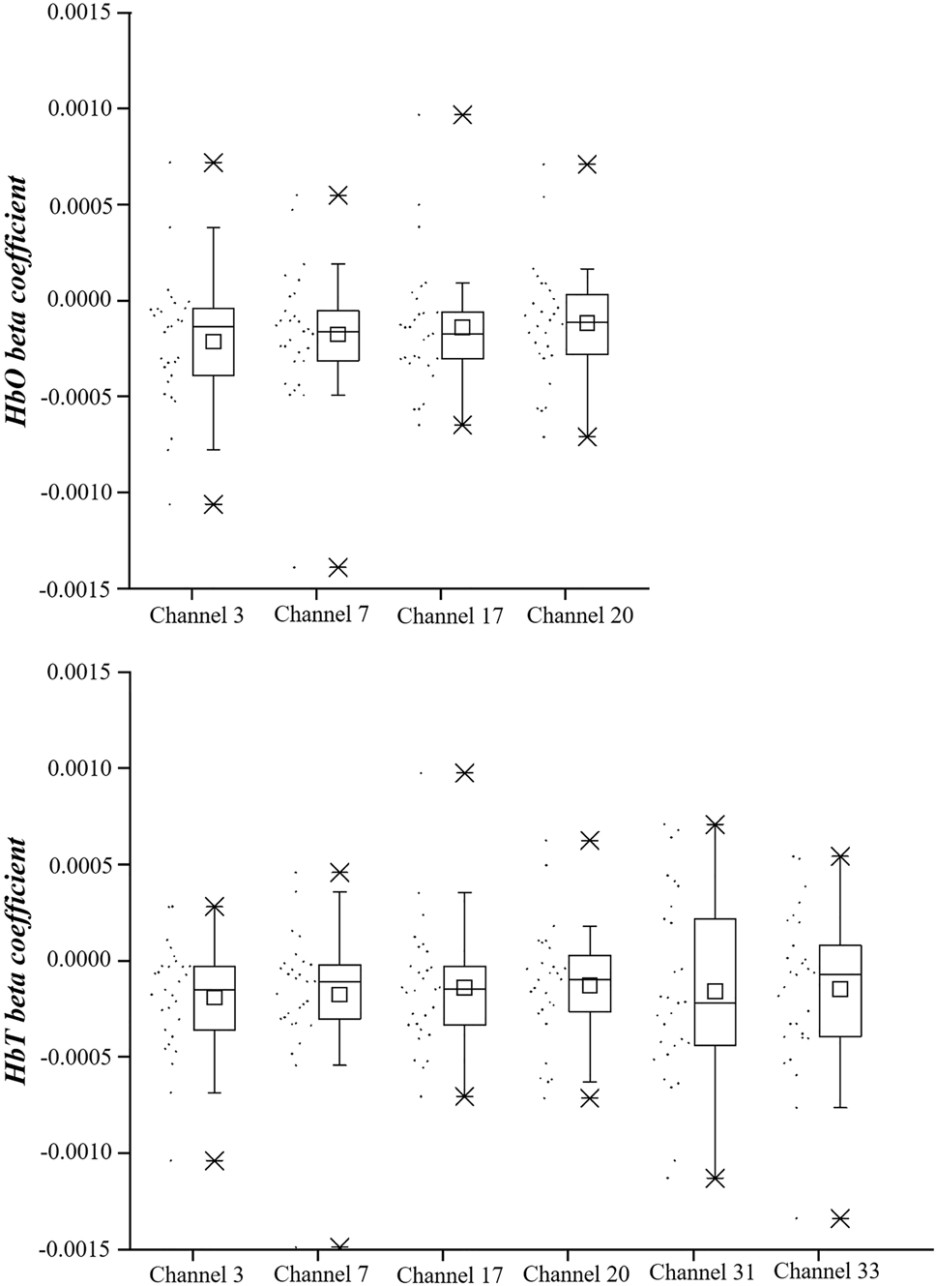
Beta coefficient of oxyhemoglobin and total hemoglobin values.

**Figure 5:**
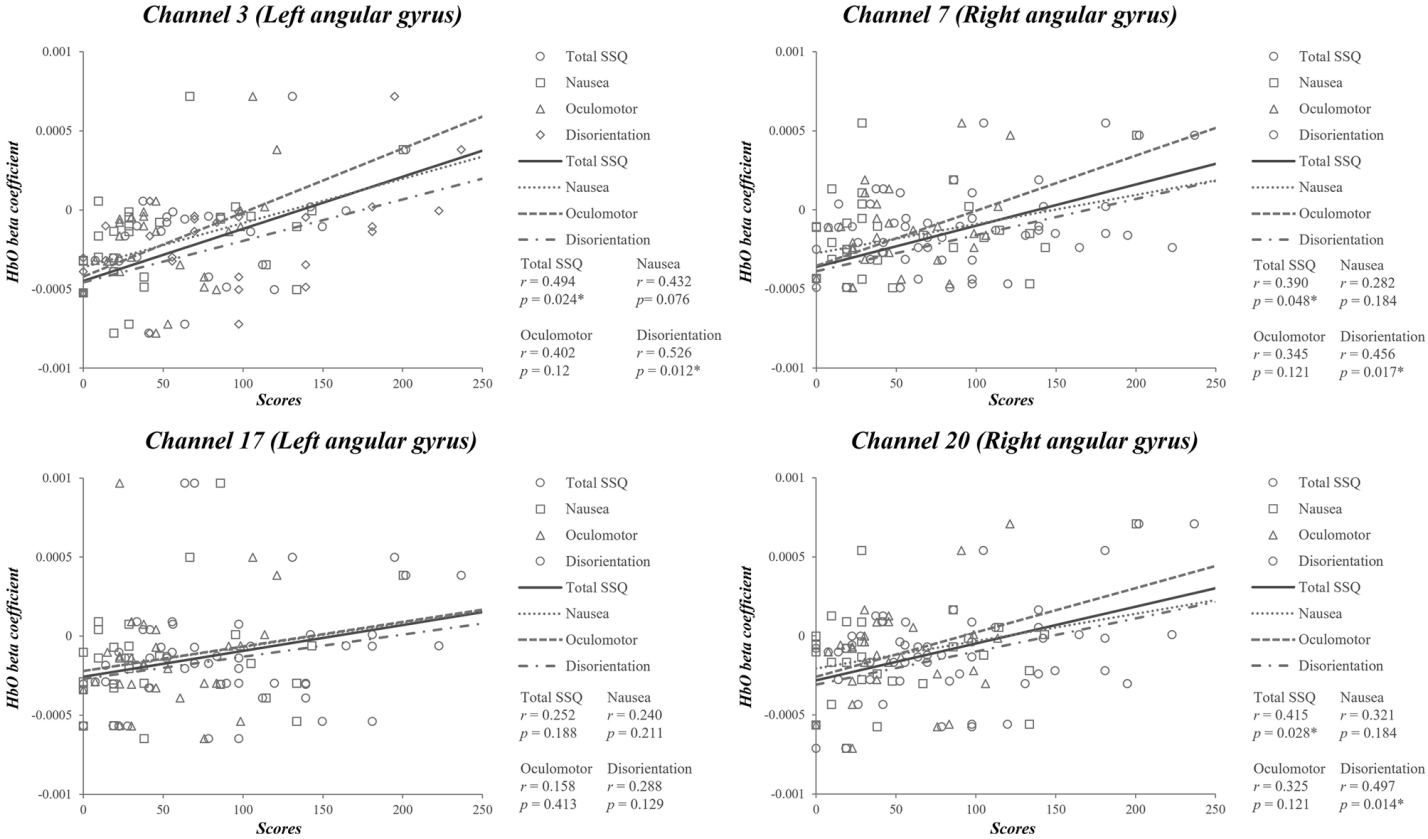
Correlation between beta coefficient of oxyhemoglobin (HbO) and Simulator Sickness Questionnaire (SSQ) scores. *Significant correlation (*p* < 0.05) with FDR correction (*p*_*corrected*_ < 0.05).

Figure 4 showed the variance of the beta coefficients of HbO and HbT. In the beta coefficient of HbO, channel 3 in the left angular gyrus showed a significant positive correlation with the total SSQ score (*r* = 0.494, *p*_*corrected*_ = 0.024) and disorientation (*r* = 0.526, *p*_*corrected*_ = 0.012). The right angular gyrus had a significant positive correlation with the total SSQ score (channel 7, *r* = 0.390, *p*_*corrected*_ = 0.048; channel 20, *r* = 0.415, *p*_*corrected*_ = 0.028) and disorientation (channel 7, *r* = 0.456, *p*_*corrected*_ = 0.017; channel 20, *r* = 0.497, *p*_*corrected*_ = 0.014) (Table 2 and Fig. 5).

**Table 2:** Correlation between beta coefficient and Simulator Sickness Questionnaire scores.

Brain region	Channel	Total SSQ		Nausea		Oculomotor		Disorientation	
		*r*	*p*_*corrected*_	*r*	*p*_*corrected*_	*r*	*p*_*corrected*_	*r*	*p*_*corrected*_
HbO									
Lt angular gyrus (BA 39)	3	0.494	0.024*	0.432	0.076	0.402	0.12	0.526	0.012*
	17	0.252	0.188	0.240	0.211	0.158	0.413	0.288	0.129
Rt angular gyrus (BA 39)	7	0.390	0.048*	0.282	0.184	0.345	0.121	0.456	0.017*
	20	0.415	0.028*	0.321	0.184	0.325	0.121	0.497	0.014*
HbT									
Lt angular gyrus (BA 39)	3	0.417	0.041*	0.401	0.062	0.284	0.237	0.453	0.02*
	17	0.150	0.436	0.165	0.392	0.060	0.757	0.179	0.353
Lt middle temporal gyrus (BA 21)	31	0.455	0.041*	0.480	0.048*	0.241	0.251	0.509	0.01*
Rt angular gyrus (BA 39)	7	0.359	0.067	0.235	0.264	0.327	0.237	0.440	0.02*
	20	0.417	0.041*	0.302	0.179	0.326	0.237	0.524	0.01*
Rt middle temporal gyrus (BA 21)	33	0.444	0.041*	0.421	0.062	0.269	0.237	0.519	0.01*

The p-value was corrected by FDR < 0.05. *BA* Brodmann area, *Lt* left, *Rt* right, *SSQ* Simulator Sickness Questionnaire, *HbO* oxyhemoglobin, *HbT* total oxyhemoglobin.

**Figure 6:**
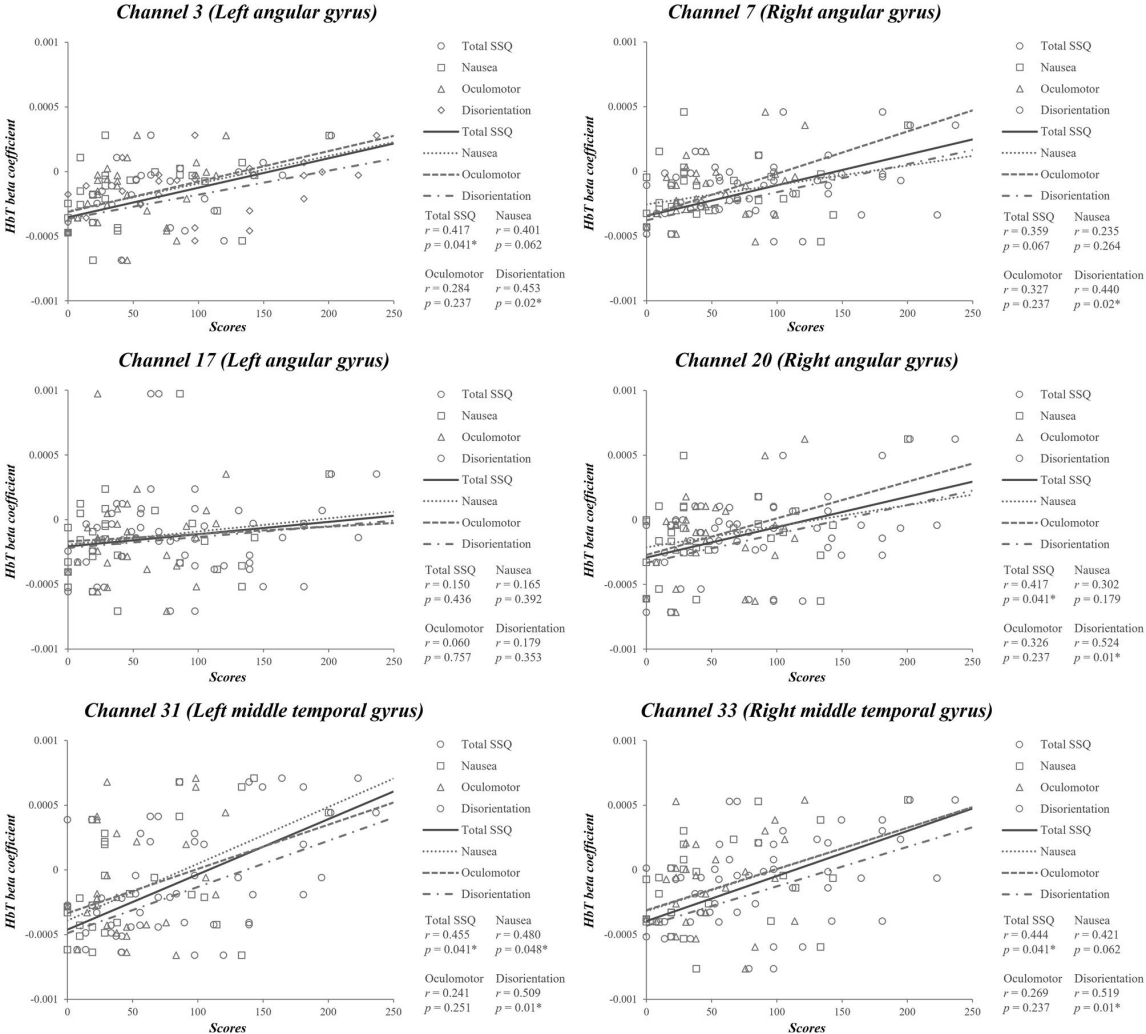
Correlation between beta coefficient of total hemoglobin (HbT) and Simulator Sickness Questionnaire (SSQ) scores. *Significant correlation with FDR correction (*p*_*corrected*_ < 0.05).

For the beta coefficient of HbT, channel 3 in the left angular gyrus had a significant positive correlation with the total SSQ score (*r* = 0.417, *p*_*corrected*_ = 0.041) and disorientation (*r* = 0.453, *p*_*corrected*_ = 0.02). The right angular gyrus had a significant positive correlation with the total SSQ score (channel 20, *r* = 0.417, *p*_*corrected*_ = 0.041) and disorientation (channel 7, *r* = 0.440, *p*_*corrected*_ = 0.02; channel 20, *r* = 0.524, *p*_*corrected*_ = 0.01). The bilateral temporal gyrus had a significant positive correlation with the total SSQ score (channel 31, *r* = 0.455, *p*_*corrected*_ = 0.041; channel 33, *r* = 0.444, *p*_*corrected*_ = 0.041) and disorientation (channel 31, *r* = 0.509, *p*_*corrected*_ = 0.01; channel 33, *r* = 0.519, *p*_*corrected*_ = 0.01) (Table 2 and Fig. 6).

## Discussion

This study investigated how the brain responds during cybersickness in healthy adults using fNIRS. Additionally, we analyzed the relationship between cortical activity based on hemodynamic responses and cybersickness symptoms. The main findings were: (1) the bilateral angular gyrus was deactivated during cybersickness in the group analysis of HbO and HbT; (2) the HbO and HbT beta coefficients in the bilateral angular gyrus had a significant positive correlation with the total SSQ and disorientation scores. These results indicated that the angular gyrus was associated with cybersickness symptoms in a virtual reality environment.

The group analysis of HbO and HbT levels showed deactivation in the bilateral angular gyrus during cybersickness. The visual-vestibular conflicts occur in the virtual reality environment because visual signals provide the illusion of movement, whereas the vestibular system lacks the corresponding linear and angular velocity for movement^35^. To resolve sensory conflicts, the brain adjusts the sensory weight toward a more reliable sensory system^22^. Specifically, the more reliable the sensory signal, the more weight is assigned (up-weighting); in contrast, less weight is assigned (down-weighting)^36^. Gallagher and Ferre reported that sensory re-weighting, which involves up-weighting of visual signals and down-weighting of vestibular signals, is likely to be a process to reduce visual-vestibular conflicts and alleviate symptoms of cybersickness^35^. This process involves reciprocal visual-vestibular inhibitory systems that predominantly extract self-motion from visual signals^35,37^. Functional neuroimaging studies have investigated reciprocal visual-vestibular inhibitory patterns during optokinetic stimulation^38,39^. Activation in the visual cortex, with deactivation of the parietoinsular vestibular cortex was observed^40^. They suggest that this pattern reflects reciprocal visual-vestibular inhibition as a multisensory mechanism for self-motion perception^38,39^. The angular gyrus in the temporoparietal junction interacts with the parietoinsular vestibular cortex, which is core region of vestibular and multisensory processing^22^. In addition, this region plays a role in vestibular processing and visual-vestibular integration^41–43^. Therefore, deactivation in the bilateral angular gyrus would be associated with the down-weighting of vestibular signals to reduce visual-vestibular conflicts and consequently alleviate cybersickness.

The HbO and HbT beta coefficients in the left angular gyrus (channel 3) positively correlated with the total SSQ score, nausea, oculomotor, and disorientation scores. In addition, the total SSQ score and disorientation positively correlated with the HbO and HbT beta coefficients in the right angular gyrus (channel 7). These results suggest that the degree of sensory reweighting in the angular gyrus affects cybersickness intensity. A multimodal magnetic resonance imaging study investigated the functional connectivity related to motion sickness susceptibility^44^. Individuals who were resistant to motion sickness demonstrated greater negative functional connectivity between the left vestibular and visual cortices than those who were susceptible to motion sickness^44^. They suggested that reciprocal visual-vestibular interactions are associated with motion sickness susceptibility. In addition, a transcranial direct current stimulation study demonstrated that the application of cathodal inhibitory stimulation to the left parieto-insular vestibular cortex (P3 international 10–20 EEG systems, electrode size 25 cm^2^) resulted in increased tolerance to nausea during motion sickness and decreased recovery time^45^. They suggested that inhibition of vestibular cortical activity delays motion sickness onset in healthy adults^45^. Based on previous studies, our findings suggest that cybersickness susceptibility is related to the degree of vestibular system down-weighting in virtual reality environments. In addition, considering the correlation coefficient, the left angular gyrus was more closely associated with cybersickness than the right angular gyrus.

We failed to detect activation or deactivation during cybersickness based on the HbR in the group analysis. This result can be explained via two perspectives. First, HbR had lower signal-to-noise ratio and reliability compared with HbO and HbT^33,46^. Second, the canonical HRF in the present study does not reflect the differences in temporal characteristics between HbO and HbR. Previous studies demonstrated that the HbR exhibited a delayed peak latency in comparison to HbO^47,48^. Given the variations in in hemodynamic responses, it would be inappropriate to apply the same canonical HRF as a regressor for both hemoglobin parameters^49^. In addition, Uga et al. suggested that the adaptive HRF approach that consider the temporal characteristics of HbR can enhance the statistical power of HbR^50^. Therefore, adaptive HRF approaches should be utilized in future studies to increases the statistical power of HbR.

## Conclusion

We demonstrated that the angular gyrus was deactivated in virtual reality environments to reduce visual-vestibular conflicts. In addition, cortical activity in the angular gyrus was associated with cybersickness intensity. These results provide an understanding of the neural mechanisms underlying cybersickness symptoms. However, this study had several limitations. First, it is difficult to generalize the results of the current study to other age groups because the participants were healthy adults in their 20 s. Second, our study employed discrete cosine transform temporal parameter with a high-pass period cutoff of 128 s, which is comparable to the duration of the task block, due to the methodological issue. Third, short-distance channels were not used, which is a promising method for correcting fNIRS signals^51,52^. Fourth, the duration of virtual reality exposure could affect cybersickness symptoms^18,53^. The duration of the exposure varied between experiments in the previous cybersickness studies (e.g., from 6 s to an hour)^18^. 10–20 min of exposure lead to most compelling symptoms of cybersickness^53^. Future studies should apply short-distance channels to improve the quality of fNIRS signals and consider the effect of various exposure times.

## Data Availability

The datasets used and/or analyzed during the current study available from the corresponding author on reasonable request.
